# Influence of age-adjusted shock index trajectories on 30-day mortality for critical patients with septic shock

**DOI:** 10.3389/fmed.2025.1534706

**Published:** 2025-05-09

**Authors:** Suru Yue, Xuefei Hou, Yingbai Wang, Zihan Xu, Xiaolin Li, Jia Wang, Shicai Ye, Jiayuan Wu

**Affiliations:** ^1^Clinical Research Service Center, Affiliated Hospital of Guangdong Medical University, Zhanjiang, Guangdong, China; ^2^Guangdong Engineering Research Center of Collaborative Innovation of Clinical Medical Big Data Cloud Service in Western Guangdong Medical Union, Affiliated Hospital of Guangdong Medical University, Zhanjiang, Guangdong, China; ^3^Department of Gastroenterology, Clinical Research Service Center, Affiliated Hospital of Guangdong Medical University, Zhanjiang, Guangdong, China

**Keywords:** septic shock, age-adjusted shock index, latent category trajectory model, critical care patients, mortality risk

## Abstract

**Background:**

Septic shock poses a high mortality risk in critically ill patients, necessitating precise hemodynamic monitoring. While the age-adjusted shock index (ASI) reflects hemodynamic stability, the prognostic value of its dynamic trajectory remains unexplored. This study evaluates whether dynamic 24-h ASI trajectories predict 30-day mortality in septic shock patients.

**Methods:**

This retrospective cohort study extracted data from the MIMIC-IV (derivation cohort, *n* = 2,559) and eICU-CRD (validation cohort, *n* = 2,177) databases. The latent category trajectory model (LCTM) classified ASI changes within 24 h of intensive care unit (ICU) admission. The association between ASI trajectory categories and 30-day mortality was evaluated using Kaplan-Meier (KM) method and Cox proportional-hazard models, reported as hazard ratios (HRs) and 95% confidence intervals (CIs).

**Result:**

Three distinct ASI trajectories were explored: persistently low (Classes 1), initial high ASI sharply decreasing followed by instability (Classes 2), and steady ASI increase (Classes 3). KM curve revealed significantly higher 30-day mortality in Class 2 (32.1%) and Class 3 (38.7%) than Class 1 (12.3%) (*P* < 0.001). After fully adjusting for covariates, Class 2 (HR = 1.68, 95% CI: 1.25–2.25, *P* = 0.001) and Class 3 (HR = 1.87, 95% CI: 1.26–2.77, *P* = 0.002) showed elevated mortality risks in the derivation cohort. Validation cohort results were consistent (Class 2: HR = 1.92, 95% CI: 1.38–2.68, *P* = 0.001) and (Class 3: HR = 1.66, 95% CI: 1.09–2.54, *P* = 0.019). Triple-robust analyses and subgroup analyses confirmed the reliability of the results.

**Conclusion:**

Dynamic 24-h ASI trajectories independently predict 30-day mortality in patients with septic shock, with unstable or rising patterns signaling high-risk subgroups. This underscores the clinical utility of real-time ASI monitoring for early risk stratification and tailored intervention.

## Introduction

Sepsis and septic shock have been declared a global health priority by the World Health Organization due to their escalating disease burden and multifactorial complexity in pathophysiological, genetic, and clinical presentation (1). In 2017, sepsis affected 48.9 million individuals worldwide, causing 11.0 million deaths, accounting for 20% of all global mortality (2). Despite advances in critical care, septic shock remains lethal, with in-hospital mortality rate exceeding 38% (3, 4). Delayed diagnosis and inadequate early interventions are key contributors to poor prognosis, underscoring the urgent need for dynamic risk stratification tools (1, 5).

Hemodynamic monitoring is essential to septic shock management. While the Surviving Sepsis Campaign (SSC) guidelines emphasize normalizing traditional markers, such as heart rate, blood pressure (BP), central venous pressure, and lactic acid (6), these static parameters often fail to predict outcomes due to individual variability in age, comorbidity, and physiological compensatory mechanism (7). For instance, normalization of BP may not reflect resolved microcirculatory dysfunction, leaving mortality unaddressed (8). This gap highlights the demand for integrative predictors that capture physiological complexity.

The Shock Index (SI), calculated from heart rate and systolic blood pressure (SBP), has emerged as a superior prognostic tool over isolated vital signs in sepsis, heart failure, trauma, myocardial infarction, and acute coronary syndrome (9–12). Gupta et al. suggested that SI has advantages over traditional vital signs in assessing higher levels of care and mortality in severe sepsis or shock (7). A retrospective study revealed that the SI trajectories within 24 h had a higher prognostic value than the baseline SI in patients with sepsis (10). However, SI's accuracy in elderly populations is limited by age-related hemodynamic changes, such as lower heart rate and higher BP (13). To address this, the age-adjusted SI (ASI) was developed. ASI outperforms SI in trauma cohorts, particularly for geriatric patients (11, 14, 15), as it accounts for age-dependent physiological decline. Despite its promise, existing ASI studies rely on static measurements, ignoring dynamic fluctuations during critical illness (16, 17). Static models suffer from “calibration drift”—declining accuracy over time due to unaccounted longitudinal changes (18).

Dynamic prediction modes using longitudinal trajectories have recently gained traction in critical care. Zhang et al. found that the trajectory of urine output within 24-h had a vital predictive value on acute kidney injury in septic patients (19). Another study found that the trajectories of SI outperformed baseline SI in prognostic prediction among septic patients (10). Yet, no studies have explored ASI trajectories in septic shock—a population where age and hemodynamic instability synergistically elevate risk. This study bridges this gap by evaluating the prognostic utility of 24-h ASI trajectories derived from high-frequency ICU data, aiming to identify high-risk hemodynamic phenotypes for early targeted intervention.

## Methods

### Data source

This multi-center retrospective cohort study utilized two large critical care medical databases: Medical Information Mart for Intensive Care IV (MIMIC-IV, derivation cohort) and eICU Collaborative Research Database (eICU-CRD, validation cohort). The most recent MIMIC-IV database (version 2.2) was released in January 2023 and contained detailed medical information for over 380,000 patients treated at Beth Israel Deaconess Medical Center between 2008 and 2019 (20). The eICU-CRD database (version 2.0), recently released in May 2018, collected a large amount of high-quality clinical information from more than 200,000 critically ill patients admitted to 208 hospitals across the United States in 2014 and 2015 (21). Because this study was conducted based on two anonymous publicly available databases, ethical approval and informed consent was waived by the Massachusetts Institute of Technology and Beth Israel Deaconess Medical Center. The author has completed the Collaborative Institutional Training Initiative and passed the National Institutes of Health examination (No.9983480), authorizing use the MIMIC-IV and eICU-CRD database.

### Participant selection

Structured query language (SQL) tool 11.2.7.0 was used to extract data from the MIMIC-IV and eICU-CRD databases. According to the sepsis-3 (22), septic shock was defined as the need for vasopressor therapy to maintain mean arterial pressure of 65 mmHg or greater and to have serum lactate levels >2 mmol/l persisting after fluid resuscitation. In this study, the International Classification of Diseases codes 9 (78,552) and 10 (R6521) were used to identify all cases diagnosed with septic shock.

The exclusion criteria were (1) patients who died within 24 h after ICU admission; (2) aged under 18 or over 89; (3) missing continuous ASI records within 24 h; and (4) patients with atrial fibrillation, ventricular arrhythmia, use of pacemaker. For patients with multiple ICU admissions, only the first admission was collected in the analysis.

### Exposure and outcome

ASI was calculated using the following equation: ASI = age (years) × [heart rate (beat/min)/SBP (mmHg)]. Previous analyses found that as the ASI increases, the prognosis of critically ill patients becomes worse (23). To fit clinical realism and data completeness, ASI was calculated as the maximum value within 3-h intervals, accommodating variable monitoring frequencies while minimizing selection bias. The survival outcome of this study was the 30-day mortality after ICU admission.

### Covariates

The covariables included demographics, treatment approaches, vital signs, laboratory tests, clinical scores, and comorbidities within 24 h after ICU admission. Demographic features included age, sex, race, body mass index (BMI), and ICU admission units. Vital signs included heart rate, SBP, and urine output. Clinical scores included the Glasgow Coma Scale (GCS) and Acute Physiological Score (APS) III score. Treatment approaches included the use of ventilator and vasopressor. Laboratory test features included glucose, hemoglobin, sodium, lactate, blood urea nitrogen (BUN), platelets, creatinine, white blood cell (WBC), calcium, hydrogen ion concentration (pH), potassium, international normalized ratio (INR), and partial prothrombin time (PPT). Comorbidities included myocardial infarction, hypertension, diabetes, liver diseases, chronic pulmonary disease, and malignant cancer. Because the eICU-CRD database did not provide information on comorbidities, data on comorbidity were only extracted from the MIMIC-IV. We used the “mice” package of R software to deal with missing values of covariables through multiple imputations (24, 25). Supplementary Table 1 provides comprehensive information on the extent of missing data and outlier rates for each covariate prior to imputation. Notably, the parameters were averaged if multiple measurements were taken within 24 h of ICU admission. To reduce information bias, we excluded variables with a missing ratio of over 20%.

Furthermore, database records will inevitably have outliers. In our study, variable values beyond the upper quartile + 1.5 × inter-quartile range (IQR) or the lower quartile – 1.5 × IQR were defined as outliers. We used the “plyr” package of R software to deal with outliers of covariables, of which the rule was that when the outlier was greater than the upper bound and lower than the lower bound, it was assigned to the upper and lower quartiles, respectively.

### Latent class trajectory model

We applied the latent class trajectory model (LCTM) to classify the 24-h ASI trajectories. LCTM is a robust approach for analyzing longitudinal data, aiming to identify distinct individual classes based on similar progression over time or age in the determinant, thereby transforming heterogeneous populations into more homogeneous pattern or class. Compared to studies that assess exposure at a single time point, LCTM has three advantages: (1) it enables in-depth phenotypic analyses of certain “high-risk” subpopulations, enhancing our understanding of etiological associations; (2) it provides a public health strategy for the early identification of varying adverse trajectories that can serve as intervention targets; and (3) it treats trajectories as outcomes, thereby offering insight into inter-individual differences (26).

A critical step of the LCTM is determining the optimal number of latent classes, assessed through the following criteria: (1) the average posterior probability (AvePP) should not be < 80%; (2) the model with the lowest Bayesian Information Standard (BIC), Akaike Information Standard (AIC), and sample-adjusted information criteria (SABIC) were selected; (3) the model's goodness-of-fit is evaluated via the highest log-likelihood ratio and entropy; (4) entropy value should not be lower than 0.9; (5) the sample size of each category must constitute at least 1% of the total sample; and (6) the simplicity and clinical interpretability of the trajectory categories are also fully considered (18, 27).

### Statistical analysis

Continuous variables were assessed for normality and expressed as mean ± standard deviation (SD) or median and interquartile range (IQR). Categorical variables were reported as numbers and percentages (%). For comparing between-group differences, the Wilcoxon rank-sum test was used for continuous variables, while the chi-squared test or Fisher's exact test was used for categorical variables. Supplementary Table 2 shows the results of the normality test for continuous variables.

The Kaplan-Meier (KM) method was used to plot the survival curves for 30-day mortality, and the log-rank test was applied to compare risk differences among trajectory classifications. We constructed five Cox proportional-hazard models to analyze the effect of trajectory changes on prognosis, reporting hazard ratios (HRs) and 95% confidence intervals (CIs). Model 1 included only the ASI trajectory classes. Model 2 was adjusted for age, sex, race, BMI, and ICU admission units. Model 3 further adjusted for APSIII and GCS in addition to the covariates in Model 2. Building upon Model 3, Model 4 included additional adjustments for glucose, hemoglobin, sodium, lactate, BUN, platelets, creatinine, WBC, calcium, pH, potassium, PT, PPT, and urine output. Finally, Model 5 represented a fully adjusted model, incorporating the use of ventilator and vasopressors, along with the adjustment for Charlson comorbidity index in the derivation cohort.

Moreover, we employed a triple-robust estimation approach to evaluate the independent correlation between ASI trajectories and prognosis in patients with septic shock. Propensity scoring models were established using multinomial logical regression and Extreme Gradient Boosting (XGBoost). Initially, the inverse probability of treatment weighting (IPTW) method estimated a propensity score (PS) for each patient based on multiple confounders. Subsequently, the PS was converted into weights to generate two IPTW cohorts, resulting in a pseudo population whose covariate distribution is independent of trajectory classes (19). However, while IPTW effectively balances confounding factors between the treatment and control groups, the resulting weighted virtual population often exceeds the original sample size, increasing the risk of false positives. To mitigate this, we also adopt stabilized IPTW (sIPTW) to limit the virtual population size and reduce false positive occurrences (28). IPTW is a widely-used statistical analysis method for sensitivity analysis, especially addressing bias due to confounding variables by calculating a weight for each subject that reflects the inverse of the probability of receiving the treatment actually administered (29). These weights were incorporated into the analyses to minimize the effects of observed confounding. This weighting strategy creates a pseudo-population wherein treatment probability is independent of measured covariates, facilitating baseline equalization between groups and augmenting the reliability of causal inference akin to those achieved in randomized controlled trials.

Furthermore, we constructed a propensity scoring model using XGBoost, an integrated machine learning algorithm based on decision tree, which excels in handling linear, non-linear, and interactive relationships between variables and covariates (30). Similarly, the KM curves were plotted in the propensity scoring models, and a log-rank test was performed. Multivariate Cox regression analyses, adjusted for all covariates, were performed on the weighted cohorts, thus achieving a comprehensive triple-robust analysis.

Subgroup analyses were performed based on age (< 65 and ≥65 years), gender (male and female), care unit (MICU/SICU, CCU, and others), and the use of ventilator and vasopressor (both no and yes). Potential interactions were assessed by incorporating cross-product terms of trajectory categories with the aforementioned covariates within the model. A two-tailed *P*-value of < 0.05 was considered statistically significant. All the statistical analyses were performed using the R software (4.2.2).

## Results

### LCTM analysis

**Figure 1:**
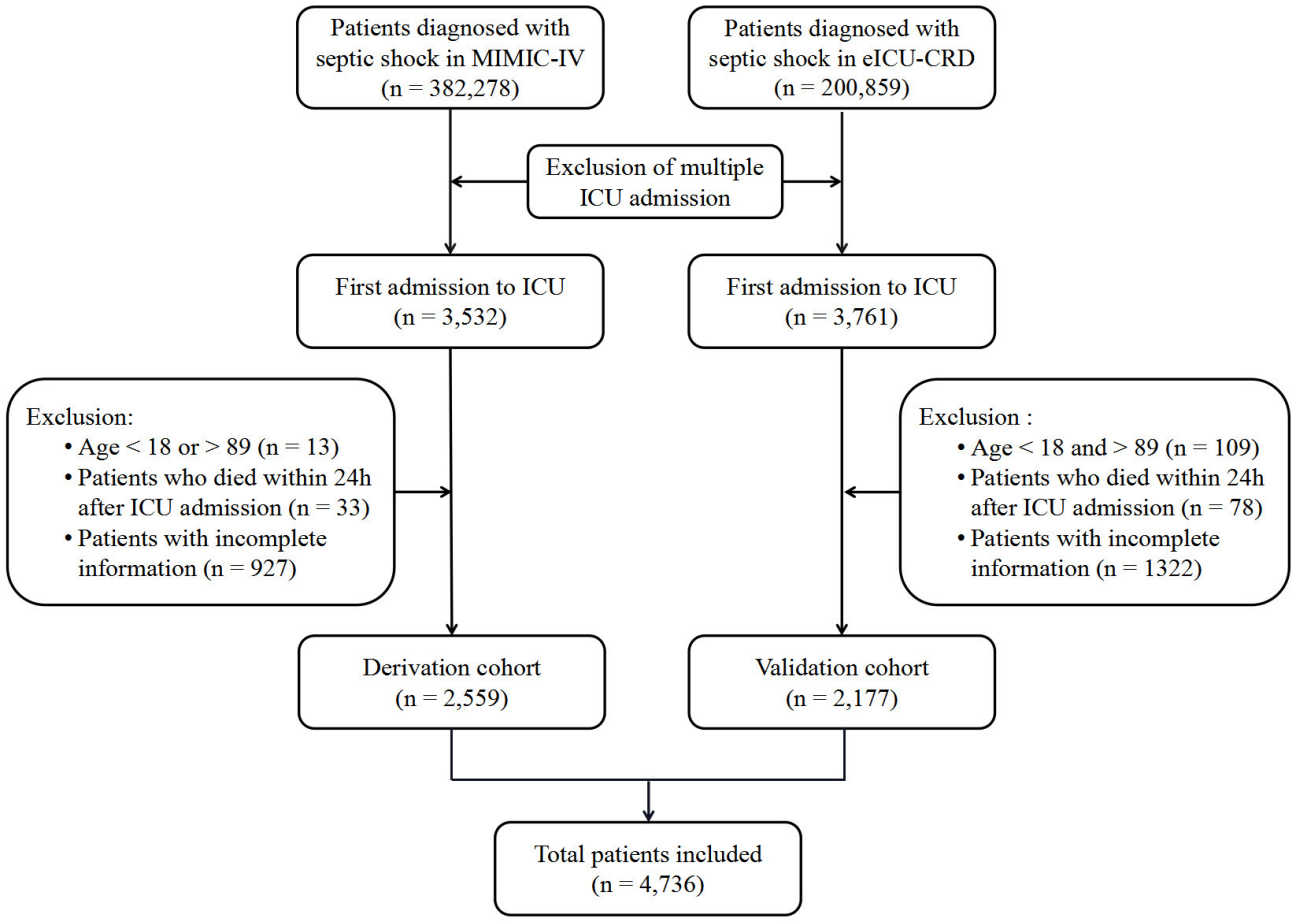
The flow chart of participant selection. MIMIC, Medical Information Mort for Intensive Care; eICU-CRD, eICU Collaborative Research Database; ICU, intensive care unit; ASI, age-adjusted shock index.

A total of 3,532 and 3,761 patients with septic shock were extracted from the MIMIC-IV and eICU-CRD databases, respectively. Following the application of exclusion criteria, 973 patients and 1,584 cases were excluded, respectively (Figure 1). Ultimately, 2,559 patients were included in the derivation cohorts, while 2,177 patients were included in the validation cohort.

Table 1 shows the results of ASI trajectory classification for both cohorts. In the derivation cohort, a trend of gradual decline in the AIC, BIC, and SABIC was showed as the categories progressed from Class 1 to Class 4, accompanied by an increase in the log-likelihood. However, the entropies showed a different trend: the entropy for the Class 3 model was higher than those of Class 2 and Class 4, yet lower than that of Class 1. The entropy values for trajectory Class 1 through 4 were 1, 0.958, 0.961, and 0.946, respectively. The sample proportion in Class 3 not only met the predefined minimum standard (1.798%) but also the AIC, BIC, SABIC, and log-likelihood values that were only slightly inferior to those of Class 4 models. Consequently, Class 3 models were determined to offer the best balance between parsimoniousness and clinical interpretability. Similar findings were corroborated in the validation cohort, leading to the conclusion that Class 3 represented the optimal classification.

**Table 1:** Statistics for choosing the best number of classes in two cohorts.

**Cohorts**	**Number of classes**	**Log likelihood**	**AIC**	**BIC**	**SABIC**	**Entropy**	**% Class 1**	**% Class 2**	**% Class 3**	**% Class 4**	**% Class 5**
Derivation	1	−44,428.09	88,870.18	88,911.11	88,888.87	1.00	100.00				
	2	−43,965.87	87,955.73	88,025.90	87,987.77	0.958	96.60	3.40			
	3	−43,761.54	87,557.09	87,656.49	87,602.48	0.961	94.76	3.44	1.80		
	4	−43,660.18	87,364.36	87,493.00	87,423.10	0.946	92.03	1.68	2.31	3.99	
	5	−43,599.90	87,253.81	87,411.69	87,325.90	0.703	48.22	44.35	1.76	2.19	3.48
Validation	1	−41,033.16	82,080.33	82,120.47	82,098.23	1.00	100.00				
	2	−40,669.36	81,362.72	81,431.54	81,393.41	0.943	96.59	3.41			
	3	−40,404.71	80,843.41	80,940.90	80,886.89	0.924	91.82	4.90	3.28		
	4	−40,280.66	80,605.31	80,731.47	80,661.57	0.686	66.58	28.17	3.15	2.10	

AIC, Akaike information criterion; BIC, Bayesian information criteria; SABIC, sample-adjusted information criteria.

**Figure 2:**
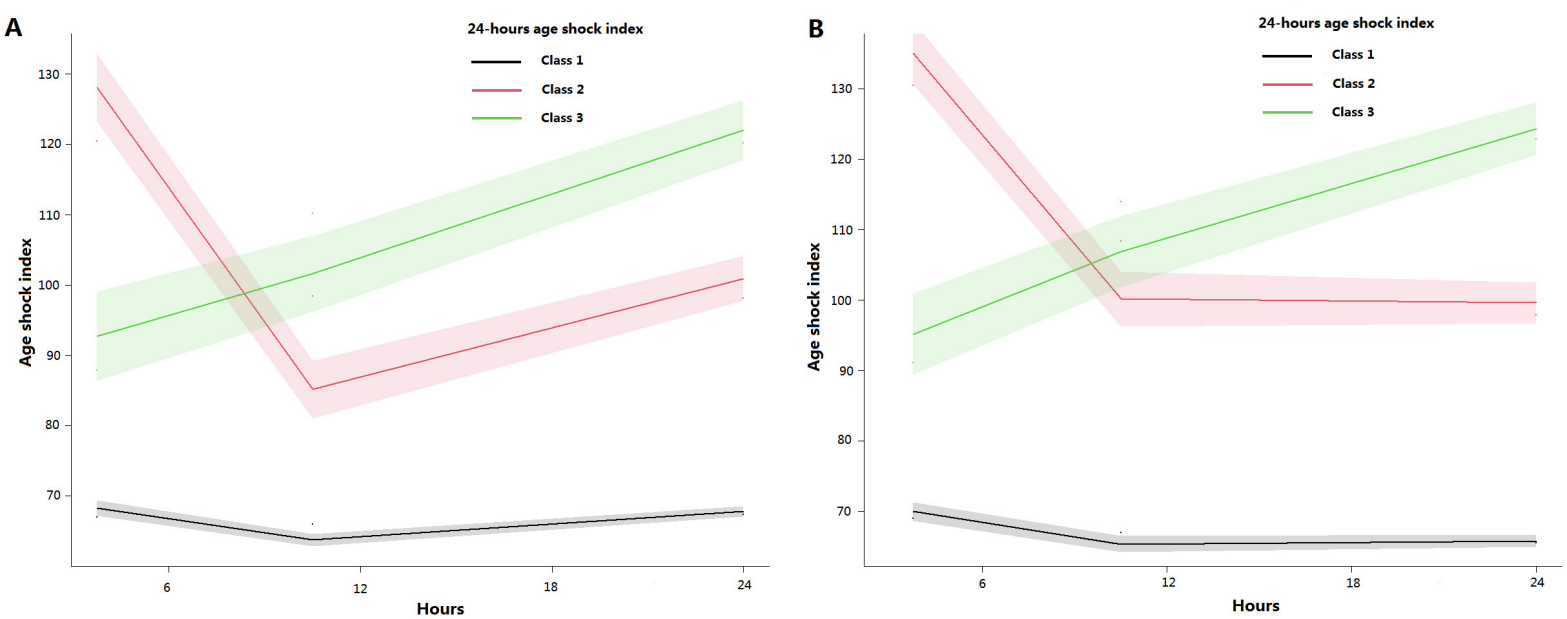
The 24 h ASI trajectories in patients with septic shock. **(A)** The derivation cohort; **(B)** The validation cohort. ASI, age-adjusted shock index.

Figure 2 illustrates the trajectories of the 24-h ASI across the three classes. The ASI trajectories were consistent between the derivation and validation cohorts. Class 1 comprised 94.76% and 91.82% of the total populations in the derivation and validation cohorts, respectively, with ASI values remaining low (< 70). Class 2 accounted for 3.44% and 4.90%, with ASI values initially high (>125), sharply decreasing to below 100, then either stabilizing or slowly increasing. Class 3 represented 1.80% and 3.28% of the cohorts, with ASI values around 95, steadily increasing to about 120.

Furthermore, the mean posterior probabilities for Class 1, Class 2, and Class 3 were 98.86%, 90.66%, and 89.21% in the derivation cohort and 97.77%, 86.30%, and 85.30% in the validation cohort, respectively (Table 2). All values exceeded 85%, confirming the reliability of the trajectory analysis results.

**Table 2:** Mean of posterior probabilities in each class.

**Cohort**	**Class**	**Probability 1**	**Probability 2**	**Probability 3**
Derivation	Class 1	0.989	0.007	0.005
	Class 2	0.092	0.907	0.002
	Class 3	0.108	0.001	0.892
Validation	Class 1	0.978	0.014	0.009
	Class 2	0.130	0.863	0.007
	Class 3	0.141	0.006	0.853

### Baseline characteristics

The baseline characteristics of the three classes are presented in Tables 3, 4. In the derivation cohort, the median age of the patients was 68.65 years, with a predominance of males (54.9%) and white (68.5%), and a median BMI of 27.3 kg/m^2^. Most patients faced comorbidities including myocardial infarction (85.3%), congestive heart failure (66.2%), cerebrovascular disease (90.9%), chronic pulmonary disease (70.1%), diabetes (66.5%), renal disease (73.0%), and liver disease (75.2%). Mechanical ventilation (88.7%) and vasopressors (80.45%) were common among this population. Compared to Class 2 and 3, patients in Class 1 were younger and exhibited lower rates of mechanical ventilation and vasopressor usage, alongside more normalized GCS score and APSIII score were.

**Table 3:** Baseline characteristics of three classes in the derivation cohort.

**Characteristics**	**Overall**	**Class 1**	**Class 2**	**Class 3**	***P*-value**
*N* (%)	2,559 (100.0)	2,425 (94.8)	88 (3.4)	46 (1.8)	
Gender (*n*, %)					0.381
Male	1,406 (54.9)	1,339 (55.2)	42 (47.7)	25 (54.3)	
Female	1,153 (45.1)	1,086 (44.8)	46 (52.3)	21 (45.7)	
Age (years)	68.7 [57.8, 80.4]	68.0 [57.4, 79.7]	79.7 [71.8, 87.6]	84.1 [71.5, 86.8]	< 0.001
Ethnicity (*n*, %)					0.980
White	1,754 (68.5)	1,663 (68.6)	61 (69.3)	30 (65.2)	
Black	356 (13.9)	336 (13.9)	13 (14.8)	7 (15.2)	
Other	449 (17.5)	426 (17.6)	14 (15.9)	9 (19.6)	
BMI (kg/m^2^)	27.3 [23.2, 32.5]	27.4 [23.3, 32.6]	25.8 [21.6, 29.7]	28.9 [24.8, 32.8]	0.007
Myocardial infarct (*n*, %)	375 (14.7)	354 (14.6)	14 (15.9)	7 (15.2)	0.938
Congestive heart failure (*n*, %)	864 (33.8)	816 (33.6)	30 (34.1)	18 (39.1)	0.737
Cerebrovascular disease (*n*, %)	233 (9.1)	219 (9.0)	5 (5.7)	9 (19.6)	0.025
Chronic pulmonary disease (*n*, %)	764 (29.9)	726 (29.9)	23 (26.1)	15 (32.6)	0.685
Diabetes (*n*, %)	858 (33.5)	812 (33.5)	29 (33.0)	17 (37.0)	0.879
Renal disease (*n*, %)	692 (27.0)	660 (27.2)	18 (20.5)	14 (30.4)	0.326
Liver disease (*n*, %)	634 (24.8)	602 (24.8)	23 (26.1)	9 (19.6)	0.684
Charlson comorbidity index	6.0 [4.0, 8.0]	6.0 [4.0, 8.0]	6.0 [5.0, 8.0]	6.0 [5.0, 8.0]	0.004
**Unit type (** ***n*** **, %)**					
MICU/SICU	2,193 (85.7)	2,081 (85.8)	73 (83.0)	39 (84.8)	0.923
CCU	139 (5.4)	131 (5.4)	6 (6.8)	2 (4.3)	
Other	227 (8.9)	213 (8.8)	9 (10.2)	5 (10.9)	
GCS	15.00 [13.00, 15.00]	15.0 [13.0, 15.0]	15.0 [13.0, 15.0]	14.00 [11.3, 15.0]	0.022
APSIII	60.00 [47.00, 78.00]	59.0 [46.0, 76.0]	73.0 [57.5, 97.0]	84.00 [64.3, 101.8]	< 0.001
Vasopressor (*n*, %)	2,057 (80.4)	1,932 (79.7)	80 (90.9)	45 (97.8)	< 0.001
Ventilation (*n*, %)	2,270 (88.7)	2,145 (88.5)	82 (93.2)	43 (93.5)	0.228
Urine output (ml)	1,220.0 [623.7, 2,100.0]	1,230.0 [649.0, 2,125.0]	857.5 [292.5, 1,682.5]	750.0 [248.0, 1,276.0]	< 0.001
BUN (mg/dL)	29.5 [19.0, 48.0]	29.0 [18.5, 48.0]	32.55 [21.9, 48.0]	38.0 [24.3, 52.4]	0.071
Calcium (mmol/l)	7.80 [7.35, 8.30]	7.85 [7.40, 8.30]	7.54 [7.24, 7.90]	7.85 [7.36, 8.39]	0.001
Chloride (mEq/l)	105.0 [100.5, 109.5]	105.0 [100.5, 109.5]	106.0 [102.4, 110.1]	105.5 [102.3, 109.5]	0.209
Creatinine (g/dl)	1.4 [1.0, 2.4]	1.4 [0.9, 2.4]	1.5 [1.0, 2.30]	2.2 [1.2, 3.2]	0.045
Glucose (mg/dl)	131.5 [107.0, 170.0]	131.0 [107.0, 169.5]	139.0 [105.6, 178.8]	136.8 [112.0, 186.3]	0.378
Bicarbonate (mEq/l)	21.0 [18.0, 23.5]	21.0 [18.0, 24.0]	19.0 [16.5, 22.6]	18.5 [16.0, 22.4]	< 0.001
Hematocrit (g/dl)	31.2 [27.7, 35.3]	31.2 [27.7, 35.3]	31.4 [28.1, 34.0]	33.1 [28.3, 37.9]	0.320
Hemoglobin (g/dl)	10.1 [8.9, 11.6]	10.1 [8.9, 11.6]	10.1 [9.0, 11.2]	10.4 [8.8, 12.2]	0.732
Platelets (10^9^/l)	187.0 [121.5, 277.5]	185.5 [121.5, 275.5]	219.8 [145.1, 318.9]	183.3 [128.0, 338.4]	0.225
Potassium (mmol/l)	4.1 [3.8, 4.7]	4.1 [3.8, 4.7]	4.2 [3.8, 4.7]	4.3 [3.9, 4.8]	0.333
WBC (10^9^/l)	13.4 [8.6, 18.8]	13.30 [8.5, 18.7]	14.27 [9.7, 21.8]	17.84 [10.4, 24.0]	0.028
Sodium (mmol/l)	138.0 [135.0, 141.0]	138.0 [135.0, 141.0]	137.3 [134.9, 141.0]	139.0 [136.0, 142.4]	0.231
INR	1.5 [1.3, 1.9]	1.5 [1.3, 1.9]	1.6 [1.3, 2.1]	1.65 [1.26, 2.49]	0.014
PTT (s)	36.4 [30.6, 46.2]	36.2 [30.5, 45.9]	40.4 [32.7, 49.9]	40.30 [33.72, 54.80]	0.004
LOS (day)	9.6 [5.5, 17.6]	9.7 [5.6, 17.8]	7.9 [2.9, 14.9]	7.3 [3.7, 11.9]	0.001
ICU duration (day)	3.8 [2.0, 8.1]	3.8 [2.0, 8.2]	2.9 [1.4, 7.2]	3.4 [1.5, 5.9]	0.017

BMI, body mass index; MICU, medical intensive care unit; SICU, surgical intensive care unit; CCU, coronary care unit; GCS, Glasgow Coma Score; APSIII, Acute Physiological Scores II; WBC, white blood cells; BUN, blood urea nitrogen; INR, International Normalized Ratio; PTT, part prothrombin time; LOS, length of hospital stay.

**Table 4:** Baseline characteristics of three classes in the validation cohort.

**Characteristics**	**Overall**	**Class 1**	**Class 2**	**Class 3**	***P*-value**
*N* (%)	2,177 (100.0)	1,995 (91.6)	115 (5.3)	67 (3.1)	
Gender (*n*, %)					0.007
Male	1,127 (51.8)	1,050 (52.6)	54 (47.0)	23 (34.3)	
Female	1,050 (48.2)	945 (47.4)	61 (53.0)	44 (65.7)	
Age (years)	67.0 [56.0, 77.0]	66.0 [55.0, 75.0]	79.0 [70.0, 84.0]	77.0 [67.5, 82.0]	< 0.001
Ethnicity (*n*, %)					0.962
White	1,829 (84.0)	1,675 (84.0)	99 (86.1)	55 (82.1)	
Black	193 (8.9)	177 (8.9)	9 (7.8)	7 (10.4)	
Other	155 (7.1)	143 (7.2)	7 (6.1)	5 (7.5)	
BMI (kg/m^2^)	26.9 [22.9, 32.7]	26.9 [22.9, 32.8]	26.9 [22.1, 33.8]	27.2 [22.6, 30.3]	0.516
**Unit type (** ***n*** **, %)**					
MICU/SICU	1,806 (83.0)	1,661 (83.3)	93 (80.9)	52 (77.6)	0.501
CCU	253 (11.6)	227 (11.4)	14 (12.2)	12 (17.9)	
Other	118 (5.4)	107 (5.4)	8 (7.0)	3 (4.5)	
GCS	14.00 [10.0, 15.0]	14.00 [10.0, 15.0]	14.00 [9.0, 15.0]	13.00 [8.0, 15.0]	0.011
APSIII	63.00 [46.0, 84.0]	62.00 [45.0, 83.0]	78.00 [59.0, 103.5]	78.00 [55.5, 98.0]	< 0.001
Vasopressor (*n*, %)	1,339 (61.5)	1,197 (60.0)	88 (76.5)	54 (80.6)	< 0.001
Ventilation (*n*, %)	793 (36.4)	709 (35.5)	49 (42.6)	35 (52.2)	0.007
Urine output (ml)	180.0 [50.0, 400.0]	180.0 [50.0, 400.0]	175.0 [62.50, 357.5]	200.0 [50.0, 390.0]	0.996
BUN (mg/dL)	27.5 [16.0, 44.5]	27.0 [15.0, 44.5]	32.5 [25.8, 44.3]	31.5 [19.5, 50.8]	0.002
Calcium (mmol/l)	7.70 [7.20, 8.20]	7.75 [7.20, 8.20]	7.65 [7.20, 8.22]	7.30 [6.93, 8.00]	0.013
Chloride (mEq/l)	51.0 [15.0, 98.0]	50.0 [15.0, 97.8]	54.5 [15.5, 97.8]	63.0 [15.0, 112.3]	0.284
Creatinine (g/dl)	1.6 [1.0, 2.6]	1.6 [0.9, 2.5]	1.8 [1.3, 2.8]	1.9 [1.4, 2.9]	0.004
Glucose (mg/dl)	112.5 [78.0, 154.5]	112.0 [78.0, 153.0]	118.5 [80.3, 167.8]	135.5 [84.0, 174.3]	0.024
Bicarbonate (mEq/l)	20.0 [15.0, 24.0]	20.5 [15.5, 24.0]	17.0 [14.0, 22.3]	17.0 [11.3, 21.5]	< 0.001
Hematocrit (g/dl)	29.1 [24.5, 34.3]	29.1 [24.5, 34.2]	27.7 [23.8, 33.2]	32.2 [26.2, 36.2]	0.019
Hemoglobin (g/dl)	8.9 [7.0, 11.4]	8.90 [7.0, 11.4]	8.75 [6.8, 11.4]	9.60 [7.3, 11.9]	0.284
Platelets (10^9^/l)	144.5 [72.0, 226.0]	144.0 [72.0, 226.0]	143.0 [58.8, 229.0]	150.5 [76.8, 215.3]	0.917
Potassium (mmol/l)	4.0 [3.6, 4.5]	4.0 [3.6, 4.5]	4.1 [3.7, 4.7]	4.0 [3.5, 4.7]	0.259
WBC (10^9^/l)	13.70 [7.4, 19.7]	13.63 [7.4, 19.5]	17.45 [9.3, 22.9]	11.80 [5.4, 17.6]	0.005
Sodium (mmol/l)	137.0 [132.0, 141.5]	137.0 [132.0, 141.5]	137.0 [133.0, 142.0]	137.0 [129.8, 143.0]	0.709
INR	1.5 [1.3, 2.0]	1.5 [1.3, 2.0]	1.6 [1.3, 2.3]	1.60 [1.3, 2.2]	0.030
PTT (s)	37.0 [31.0, 44.9]	37.0 [31.0, 44.6]	37.0 [29.7, 47.2]	39.0 [27.2, 48.8]	0.776
LOS (day)	7.78 [4.5, 13.9]	7.86 [4.6, 13.9]	5.55 [1.5, 11.6]	5.82 [2.1, 12.7]	< 0.001
ICU duration (day)	3.6 [1.9, 7.0]	3.6 [1.9, 7.1]	2.9 [1.4, 5.8]	3.4 [1.8, 8.5]	0.121

BMI, body mass index; MICU, medical intensive care unit; SICU, surgical intensive care unit; CCU, coronary care unit; GCS, Glasgow Coma Score; APSIII, Acute Physiological Scores II; WBC, white blood cells; BUN, blood urea nitrogen; INR, International Normalized Ratio; PTT, part prothrombin time; LOS, length of hospital stay.

In the validation cohort, the median age and BMI of septic shock patients were 67 years and 26.9 kg/m^2^, respectively, with a majority being males (51.8%) and white (84%). The rates of vasopressors and mechanical ventilation use were 61.5% and 36.4%, respectively. Compared to patients in Class 2 and 3, patients in Class 1 were younger, had higher GCS scores, lower APSIII scores, and reduced reliance on vasopressors and ventilators.

### KM survival analyses

**Figure 3:**
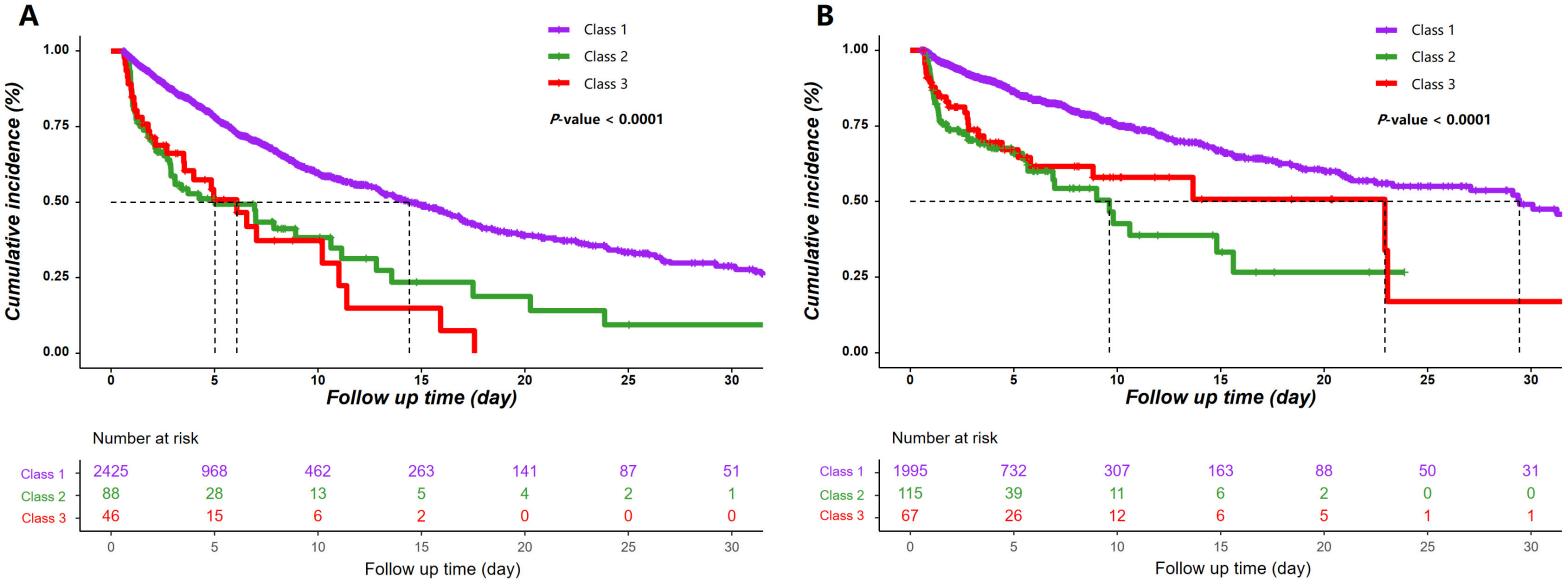
Kaplan–Meier survival curves according to the 24 h ASI trajectories. **(A)** The derivation cohort; **(B)** The validation cohort. ASI, age-adjusted shock index.

Figure 3 shows significant difference in 30-day mortality across the three ASI trajectory classes (*P* for log-rank test < 0.001). Patients in Class 1 experienced superior survival outcome compared to those in Class 2 and 3 in both the derivation and validation cohorts.

### Cox proportional-hazard regression models

The results of multicollinearity diagnoses are shown in Supplementary Table 3. None of the variance inflation factors (VIFs) exceeded 5, indicating no multicollinearity between the variables. Cox proportional-hazard models were then utilized to explore the relationship between the trajectory classes and prognosis after adjusting for various confounders, with findings reported in Table 5. In the derivation cohort, all five Cox regression models indicated that Class 3 had the highest risk of 30-day mortality, followed by Class 2, while Class 1 exhibited the lowest risk (*P* < 0.05). In Model 5, which included the most comprehensive set of covariates, Class 2 and Class 3 had 68% and 87% increased risks of 30-day mortality relative to Class 1, with HRs of 1.68 (95% CI: 1.25–2.25, *P* = 0.001) and 1.87 (95% CI: 1.26–2.77, *P* = 0.002), respectively. Similar results were observed in the validation cohort; however, Class 2 exhibited the highest mortality risk, followed by Class 3. In this fully adjusted model (Model 5), Class 2 and Class 3 were significantly associated with increased mortality risk compared to Class 1, with HRs of 1.92 (95% CI: 1.38–2.68, *P* = 0.001) and 1.66 (95% CI: 1.09–2.54, *P* = 0.019), respectively.

**Table 5:** Results of Cox proportional hazard models.

**Cohort**	**Class**	**Model 1**		**Model 2**		**Model 3**		**Model 4**		**Model 5**	
		**HR (95% CI)**	***P*** **-value**	**HR (95% CI)**	***P*** **-value**	**HR (95% CI)**	***P*** **-value**	**HR (95% CI)**	***P*** **-value**	**HR (95% CI)**	***P*** **-value**
Derivation	Class1	1 (Reference)		1 (Reference)		1 (Reference)		1 (Reference)		1 (Reference)	
	Class2	2.48 (1.88, 3.27)	< 0.001	1.99 (1.50, 2.65)	< 0.001	1.70 (1.27, 2.27)	< 0.001	1.69 (1.26, 2.26)	< 0.001	1.68 (1.25, 2.25)	0.001
	Class3	2.86 (1.96, 4.17)	< 0.001	2.57 (1.76, 3.77)	< 0.001	2.12 (1.44, 3.12)	< 0.001	2.10 (1.43, 3.09)	< 0.001	1.87 (1.26, 2.77)	0.002
Validation	Class1	1 (Reference)		1 (Reference)		1 (Reference)		1 (Reference)		1 (Reference)	
	Class2	3.22 (2.36, 2.40)	< 0.001	2.85 (2.07, 3.93)	< 0.001	2.36 (1.70, 3.27)	< 0.001	2.30 (1.66, 3.19)	< 0.001	1.92 (1.38, 2.68)	0.001
	Class3	2.42 (1.62, 3.61)	< 0.001	2.22 (1.47, 3.35)	< 0.001	1.93 (1.28, 2.92)	0.002	1.88 (1.25, 2.89)	0.003	1.66 (1.09, 2.54)	0.019

HR, hazard ratio; CI, confidence interval.

Model 1 only included the ASI trajectory classes.

Model 2 was adjusted for demographic features, including age, gender, ethnicity, BMI, and unit type.

Model 3 was additionally adjusted for clinical scores, including GCS and APSIII.

Model 4 was additionally adjusted for laboratory tests, including glucose, hemoglobin, sodium, lactate, BUN, platelets, creatinine, WBC, calcium, pH, potassium, PT, PPT, and urine output.

Model 5 was additionally adjusted for clinical therapy, including mechanical ventilation and vasopressor, and Charlson comorbidity index was also adjusted in the derivation cohort.

### Triple-robust analysis/sensitivity analysis

The baseline characteristics for the IPTW and sIPTW (from multinomial logistic regression) and the IPTW dataset (from the XGBoost algorithm) are presented in Supplementary Tables 4, 5.

In the derivation cohort, a good balance between covariates across classes was achieved following IPTW and sIPTW, with XGBoost outperforming multinomial logistic regression. After IPTW using multinomial logistic regression, however, the distribution of age, sex, vasopressor, and sodium remained unbalanced (*P* < 0.05). Following sIPTW by multinomial logistic regression, the distribution for age, weight, GCS, APSIII, urine output, BUN, calcium, chlorine, bicarbonate, hematocrit, sodium, INR, and PPT also remained unbalanced (*P* < 0.05). In contrast, after IPTW based on the XGBoost algorithm, only urine output, APSIII, and age were notably unbalanced (*P* < 0.05).

Similarly, in the validation cohort, covariates across classes were well balanced after IPTW and sIPTW, with XGBoost again demonstrated superior performance compared to multinomial logistic regression. Following IPTW using multinomial logistic regression, distribution for age, sex, urine output, and platelets remained unbalanced (*P* < 0.05). After sIPTW, the distribution for age, sex, weight, GCS, APSIII, vasopressor, machine ventilation, glucose, bicarbonate, hematocrit, hemoglobin, WBC, and INR were unbalanced (*P* < 0.05). In contrast, following IPTW based on the XGBoost algorithm, only the distribution for sex and age remained unbalanced (*P* < 0.05). Supplementary Figure 1 illustrates significant differences in 30-day mortality among the three ASI trajectory classes, as determined by triple robust estimations in both the derivation and validation cohorts (*P* for log-rank test < 0.001).

The findings from the triple-robust analysis are detailed in Table 6. The results from IPTW utilizing both XGBoost and multinomial logistic regression aligned with those observed in both the derivation and validation cohorts. According to the results from the fully adjusted multivariate Cox regression model, the risk of 30-day mortality remained elevated in Class 2 and 3 compared to Class 1 (*P* < 0.05). These consistent findings across both the original and robust datasets underpin the reliability of this study, indicating similar hazard trends.

**Table 6:** Results of subgroup analyses.

**Cohort**	**Subgroup**	***N***	**Class 1**	**Class 2**		**Class 3**		***P* for interaction**
			**HR (95% CI)**	**HR (95% CI)**	***P*** **-value**	**HR (95% CI)**	***P*** **-value**	
Derivation	All people	2,559	1 (Reference)	2.48 (1.88–3.27)	< 0.001	2.87 (1.97–4.19)	< 0.0001	
	Age (years)							0.090
	< 65	1,032	1 (Reference)	4.95 (2.33–10.52)	< 0.001	2.39 (0.89–6.43)	0.080	
	≥65	1,527	1 (Reference)	2.01 (1.49–2.71)	< 0.001	2.71 (1.79–4.08)	< 0.001	
	Gender							0.877
	Male	1,406	1 (Reference)	2.34 (1.57–3.49)	< 0.001	2.61 (1.53–4.46)	< 0.001	
	Female	1,153	1 (Reference)	2.72 (1.84–4.00)	< 0.001	3.19 (1.87–5.46)	< 0.001	
	Race							0.928
	White	1,754	1 (Reference)	2.37 (1.68–3.33)	< 0.001	3.08 (1.95–4.88)	< 0.001	
	Black	356	1 (Reference)	3.45 (1.73–6.89)	< 0.001	2.12 (0.77- 5.78)	0.140	
	Others	449	1 (Reference)	2.35 (1.20–4.61)	0.010	2.73 (1.11–6.68)	0.030	
	Care unit							0.129
	MICU/SICU	2,193	1 (Reference)	3.05 (2.26–4.13)	< 0.001	3.02 (2.01–4.55)	< 0.001	
	CCU	139	1 (Reference)	0.88 (0.21–3.63)	0.860	3.01 (0.72–12.48)	0.130	
	Others	227	1 (Reference)	1.31 (0.52–3.29)	0.570	2.07 (0.50–8.55)	0.310	
	Vasopressor							0.936
	No	501	1 (Reference)	1.39 (0.34–5.65)	0.645	0.00 (0.00–1.00)	-	
	Yes	2,012	1 (Reference)	2.65 (2.00–3.52)	< 0.001	3.19 (2.18–4.67)	< 0.001	
	Ventilation							0.900
	No	286	1 (Reference)	3.36 (1.02–11.07)	0.046	2.25 (0.30–16.72)	0.430	
	Yes	2,227	1 (Reference)	2.45 (1.84–3.26)	< 0.001	2.91 (1.98–4.28)	< 0.001	
Validation	All people	2,177	1 (Reference)	3.23 (2.37–4.41)	< 0.001	2.41 (1.61–3.60)	< 0.001	
	Age (years)							0.232
	< 65	953	1 (Reference)	4.71 (2.29–9.68)	< 0.001	1.53 (0.49–4.82)	0.47	
	≥ 65	1,224	1 (Reference)	2.65 (1.87–3.77)	< 0.001	2.35 (1.52–3.64)	< 0.001	
	Gender							0.584
	Male	1,127	1 (Reference)	2.76 (1.70–4.51)	< 0.001	2.44 (1.20–4.97)	0.010	
	Female	1,050	1 (Reference)	3.80 (2.52–5.71)	< 0.001	2.55 (1.56–4.18)	< 0.001	
	Race							0.677
	White	1,829	1 (Reference)	3.11 (2.23–4.34)	< 0.001	2.62 (1.73–3.98)	< 0.001	
	Black	193	1 (Reference)	4.23 (1.47–12.22)	0.010	0.77 (0.10–5.69)	0.800	
	Others	155	1 (Reference)	3.67 (0.82–16.4)	0.090	2.29 (0.30–17.47)	0.430	
	Care unit							0.696
	MICU/SICU	1,806	1 (Reference)	3.09 (2.19–4.37)	< 0.001	2.52 (1.61–3.93)	0.090	
	CCU	253	1 (Reference)	6.25 (2.50–15.61)	< 0.001	3.23 (1.20–8.72)	0.470	
	Others	118	1 (Reference)	2.36 (0.69–8.01)	0.170	0.00 (0.00–1.00)	< 0.001	
	Vasopressor							0.188
	No	838	1 (Reference)	2.00 (0.88–4.58)	0.100	3.39 (1.57–7.32)	< 0.001	
	Yes	1,339	1 (Reference)	3.32 (2.37–4.66)	< 0.001	2.02 (1.26–3.23)	< 0.001	
	Ventilation							0.817
	No	1,384	1 (Reference)	3.13 (1.99–4.91)	< 0.001	2.68 (1.45–4.96)	< 0.001	
	Yes	793	1 (Reference)	3.22 (2.09–4.95)	< 0.001	2.12 (1.25–3.60)	0.010	

HR, hazard ratio; CI, confidence interval; MICU, medical intensive care unit; SICU, surgical intensive care unit; CCU, coronary care unit.

### Subgroup analyses

Results from subgroup and interaction analyses are shown in Table 7. A significant correlation was noted between ASI trajectories and the risk of 30-day mortality across all subgroups. Moreover, no statistical significance was detected in cross-product terms involving the ASI trajectories and the stratified covariates, suggesting that the absence of interaction effects.

**Table 7:** Results of triple robust analyses.

**Cohort**	**Class**	**IPTW**		**Stabilized IPTW**		**XGBoost**	
		**HR (95% CI)**	***P*** **-value**	**HR (95% CI)**	***P*** **-value**	**HR (95% CI)**	***P*** **-value**
Derivation	Class 1	1 (Reference)		1 (Reference)		1 (Reference)	
	Class 2	1.68 (1.18, 2.40)	0.004	1.68 (1.18, 2.40)	0.004	2.22 (1.49, 3.31)	< 0.001
	Class 3	2.94 (2.10, 4.12)	< 0.001	2.94 (2.11, 4.12)	< 0.001	1.58 (1.13, 2.21)	0.008
Validation	Class 1	1 (Reference)		1 (Reference)		1 (Reference)	
	Class 2	2.36 (1.61, 3.45)	< 0.001	2.36 (1.62, 3.45)	< 0.001	2.00 (1.32, 3.03)	0.001
	Class 3	2.05 (1.21, 3.46)	0.007	2.05 (1.21, 3.46)	0.007	1.81 (1.05, 3.11)	0.033

HR, hazard ratio; CI, confidence interval; XGBoost, Extreme Gradient Boosting; IPTW, inverse probability of treatment weighting.

## Discussion

In this study, LCTM was used to classify the 24-h ASI trajectories following ICU admission. A key finding of this study was the significant association between ASI trajectories and the risk of 30-day mortality among patients with septic shock. These results were corroborated by analyses conducted in two large critical care databases, MIMIC-IV and eICU-CRD. Thus, dynamic monitoring of ASI and early assessment of its trajectory may aid in identifying high-risk patients with septic shock, guiding early interventions to mitigate adverse outcomes.

Our application of LCTM to analyze dynamic changes in ASI over 24 h led to the identification of three distinct hemodynamic trajectory patterns. Statistically, the three-class solution demonstrated optimal performance in terms of AIC (1,265.3), BIC (1,289.7), and SABIC (1,274.1), outperforming alternative classifications. Moreover, the AvePP for all classes exceeding 85%, accompanied an entropy of 0.92, significantly above the recommended threshold of 0.8, thereby ensuring clear class separation and individual membership certainty. These trajectory patterns and their prognostic implications were rigorously validated in both the MIMIC-IV (derivation) and eICU-CRD (validation) cohorts, reinforcing their generalizability. Clinically, these trajectories reflect the pathophysiological heterogeneity present in septic shock, categorizing patients into three profiles: persistently low ASI (Class 1), sharp decline followed by instability (Class 2), and steady ASI increase (Class 3). These findings underscore the translational value of integrating dynamic ASI monitoring into septic shock management protocols.

This study contributes new insights relative to previous studies. Our findings indicated that patients with an initial high ASI that sharply decreased and then remained stable or slowly increased (Class 2), alongside those exhibiting a steady growth of ASI (Class 3), were at an elevated risk of 30-day mortality compared to those with a persistently low ASI (Class 1). The progression and recovery from septic shock are inherently dynamic, characterized by complex relationship between physiological parameters such as BP and HR, and clinical outcomes. Notably, Class 2 and 3 maintained consistently elevated ASI levels, which might indicate acute hypovolemia and circulatory failure, ultimately contributing to increasing mortality risk (11).

In the derivation cohort, although the baseline ASI for Class 3 was not the highest, it exhibited a continuous upward trend, ultimately surpassing the other classes and leading to the highest mortality risk. Persistent severe hypotension, delayed initial fluid resuscitation, and prolonged tachycardia may underlie the sustained increase in ASI and ensuring poor prognosis for Class 3 patients. The pathophysiology of septic shock encompasses a cascade of intracellular events triggered by pathogens, affecting immune, epithelial, endothelial cells, and the entire neuroendocrine system (31, 32). The deranged and deregulated host responses observed in Class 3 are more complex and severe than those in other classes, characterized by sustained excessive inflammation, immunosuppression, and an inability to restore normal pro- and anti-inflammatory homeostasis, ultimately leading to a markedly pathological state (32–36). Inflammatory responses can impair tissues, while anti-inflammatory phenomena may result in leukocyte reprogramming and changes in the immune system (32, 37). This multifaceted process can evolve rapidly, often outpacing the effectiveness of therapeutic interventions. Moreover, organ perfusion disorders extending beyond microcirculation may drive multiple organ failures (38). Additionally, while vasoactive drugs are preferred treatment for septic shock, it has been shown that their use may induce immunosuppression, promote infection, attenuate pro-inflammatory cytokine production, and heighten anti-inflammatory cytokine levels (39, 40). Our study also found that patients in Class 3 had higher rates of vasopressor use compared to those in other classes. Therefore, regardless of high baseline ASI levels in septic shock patients, timely and effective interventions at reducing ASI and enhancing circulatory function may lower mortality risks.

In contrast to the derivation cohort, our validation cohort revealed that Class 2 posed the highest mortality risk. This discrepancy may be closely related to the older age of patients in this group. While patients in Class 3 had the oldest average age in the derivation cohort, those in Class 2 were older in the validation cohort. Age is recognized as a major risk factors affecting the prognosis of critically ill patients, particularly in incidence of septic shock in the elderly (4). Age-related decline in immune function, organ and tissue aging, and the accumulation of comorbidities increase mortality risk among elderly patients (41, 42). According to the SSC guidelines, prompt fluid resuscitation and antibiotic therapy are critical upon detection of septic shock (6). Delay in antibiotic treatment or fluid management have been linked to increased mortality risk (43). Furthermore, vasopressors are routinely used to maintain mean arterial pressure when hypotension persists despite fluid resuscitation. Paradoxically, some commonly vasoactive drugs, such as norepinephrine, may exert inhibitory effects on cellular immune function, fostering bacterial growth and subsequently elevating susceptibility to secondary infections among septic shock patients (38). As such, age and treatment factors are crucial elements influencing prognosis in this population, further reinforcing the utility of ASI over traditional severity indexes for risk assessment in elderly patients. In addition, we also observed differences in comorbidity data availability and vasopressor/ventilator use between the derivation and validation cohorts. These discrepancies are inherent to real-world data originating from diverse centers, reflecting variations in patient demographics, healthcare resources, clinical guidelines, and data collection protocols. Despite these differences, our study, leveraging a large sample and advanced statistical methods (latent class trajectory models, triple-robust analyses, and subgroup analyses), provides valuable insights into the prognostic utility of dynamic ASI trajectories.

Dynamic indices offer valuable insights into the progression of sepsis, and trajectories of markers such as 24-h urine output (19), C-reactive protein (44), SBP (45), and SI (10) have been used to predict patient outcomes. Despite this, the potential of 24-h ASI trajectories as a prognostic marker in septic shock—a condition where age and hemodynamic instability compound mortality risk—has been overlooked. Our study addresses this critical gap by being the first to examine the association between 24-h ASI trajectories and survival outcomes in patients with septic shock, offering potentially valuable insights for risk stratification and management. Moreover, our study has several notable strengths compared with previous researches. Firstly, the derivation and validation cohorts were drawn from two extensive critical care databases, MIMIC-IV and eICU-CRD, noted for their long-term, high-quality and reliable data. Secondly, the LCTM framework was performed to classify the 24-h ASI trajectories in patients with septic shock, alongside the establishment of a series of Cox regression models to adjust for various confounders. Thirdly, we employed triple-robust estimations to validate the impact of ASI trajectories on mortality risk, indicating the stability and reliability of our findings. Finally, ASI is a non-invasive, convenient, repeatable metric that can dynamically monitored comprising three readily available indexes (age, heart rate, and SBP), which may facilitate rapid identification of high-risk patients and serve as a basis for clinical decision-making, enabling early interventions to improve prognosis.

However, the study exhibits certain limitations. As a retrospective analysis, it is subject to various biases, necessitating validation via multicenter prospective studies. Additionally, some potential confounders were not accounted for due to excessive missing data. Although multiple imputations were used to address missing data, they may introduce deviations from true values. Furthermore, given that the MIMIC and eICU-CRD databases focus on critically ill patients, our investigation was limited to those in ICUs, excluding patients treated outside of this setting. While IPTW aimed to balance baseline characteristics, minor imbalances persisted across trajectory classifications. The impact of these remaining imbalances on our results warrants consideration.

## Conclusion

This study demonstrates that the 24-h ASI trajectories following ICU admission are significantly associated with the risk of short-term mortality in critically ill patients with septic shock. Patients exhibiting high baseline ASI levels were more likely to experience mortality within 30 days-regardless of whether ASI remained elevated or decreased within the subsequent 24 h). Our findings suggest that ASI trajectories could serve as an effective dynamic bedside tool for risk stratification. Clinicians should prioritize early hemodynamic optimization in patients exhibiting rising or unstable ASI trajectories to mitigate mortality risks.

## Data Availability

Data of this study were obtained from two large critical care databases, MIMIC-IV and eICU-CRD and the author (SYu) obtained permission (No.9983480). All the data can be found on its website (https://mimic.physionet.org/; https://physionet.org/content/eicu-crd/2.0/).
