# Supplementary material for: Influence of age-adjusted shock index trajectories on 30-day mortality for critical patients with septic shock
Source: Front Med (Lausanne). 2025 May 9;12:1534706. doi: 10.3389/fmed.2025.1534706 (PMC12098450; doi:10.3389/fmed.2025.1534706)
Supplement: Supplementary file 1 [file Data_Sheet_1.zip › Supplementary Material/Supplement Figure legend.docx]

**Supplement Figure 1. Cumulative survival curves after triple robust estimations.**

(A) the derivation cohort by IPTW adjustment; (B) the derivation cohort by sIPTW adjustment; (C) the derivation cohort by XGBoost adjustment; (D) the validation cohort by IPTW adjustment; (E) the validation cohort by sIPTW adjustment; (F) the validation cohort by XGBoost adjustment. IPTW, inverse probability of treatment weighting; sIPTW, stabilized inverse probability of treatment weighting; XGBoost, Extreme Gradient Boosting.
