# Supplementary material for: Influence of age-adjusted shock index trajectories on 30-day mortality for critical patients with septic shock
Source: Front Med (Lausanne). 2025 May 9;12:1534706. doi: 10.3389/fmed.2025.1534706 (PMC12098450; doi:10.3389/fmed.2025.1534706)
Supplement: Supplementary file 1 [file Data_Sheet_1.zip › Supplementary Material/Supplement Table 1.docx]

**Supplement Table 1. The scenario of missing variables before imputation.**

| Variables | MIMIC-Ⅳ | | | eICU-CRD | | |
| --- | --- | --- | --- | --- | --- | --- |
|  | Number of outliers | Number of missing values | Missing ratio | Number of outliers | Number of missing values | Missing ratio |
| Gender | - | 0 | 0% | - | 0 | 0% |
| Age | 14 | 0 | 0% | 18 | 4 | 0.18% |
| Ethnicity | - | 0 | 0% | - | 0 | 0% |
| Weight | 64 | 36 | 1.41% | 61 | 21 | 0.96% |
| Height | 0 | 203 | 7.93% | 4 | 4 | 0.18% |
| Unit type | - | 0 | 0% | - | 0 | 0% |
| GCS | 296 | 1 | 0.04% | 0 | 382 | 17.55% |
| APSIII | 40 | 0 | 0% | 29 | 192 | 8.82% |
| Vasopressor | - | 0 | 0% | - | 0 | 0% |
| Ventilation | - | 0 | 0% | - | 0 | 0% |
| Urine output | 108 | 88 | 3.44% | 124 | 354 | 16.26% |
| BUN | 115 | 2 | 0.08% | 58 | 2 | 0.09% |
| Calcium | 61 | 15 | 0.59% | 50 | 93 | 4.27% |
| Chloride | 53 | 3 | 0.12% | 0 | 69 | 3.17% |
| Creatinine | 199 | 2 | 0.08% | 138 | 68 | 3.12% |
| Glucose | 160 | 5 | 0.20% | 101 | 20 | 0.92% |
| Bicarbonate | 79 | 3 | 0.12% | 212 | 113 | 5.19% |
| Hematocrit | 28 | 5 | 0.20% | 230 | 111 | 5.10% |
| Hemoglobin | 24 | 6 | 0.23% | 377 | 110 | 5.05% |
| Platelets | 80 | 7 | 0.27% | 37 | 125 | 5.74% |
| Potassium | 40 | 3 | 0.12% | 89 | 61 | 2.80% |
| WBC | 92 | 7 | 0.27% | 60 | 131 | 6.02% |
| Sodium | 122 | 3 | 0.12% | 248 | 68 | 3.12% |
| INR | 259 | 144 | 5.63% | 101 | 139 | 6.65% |
| PTT | 202 | 162 | 6.33% | 114 | 145 | 6.85% |

MIMIC, Medical Information Mart for Intensive Care; eICU-CRD, eICU Collaborative Research Database; GCS: Glasgow Coma Score; APSIII: Acute Physiological Scores II; WBC: white blood cells; BUN: blood urea nitrogen; INR: International Normalized Ratio; PTT: part prothrombin time.
