# Supplementary material for: Influence of age-adjusted shock index trajectories on 30-day mortality for critical patients with septic shock
Source: Front Med (Lausanne). 2025 May 9;12:1534706. doi: 10.3389/fmed.2025.1534706 (PMC12098450; doi:10.3389/fmed.2025.1534706)
Supplement: Supplementary file 1 [file Data_Sheet_1.zip › Supplementary Material/Supplement Table 2.docx]

**Supplement Table 2. Results of the normality tests**

| Characteristics | MIMIC-Ⅳ | | eICU-CRD | |
| --- | --- | --- | --- | --- |
|  | *P* value | Normal Distribution | *P* value | Normal Distribution |
| Age | 0.033 | FALSE | < 0.001 | FALSE |
| BMI | 0.002 | FALSE | < 0.001 | FALSE |
| GCS | < 0.001 | FALSE | < 0.001 | FALSE |
| ASPIII | 0.014 | FALSE | 0.002 | FALSE |
| Urine output | < 0.001 | FALSE | < 0.001 | FALSE |
| BUN | < 0.001 | FALSE | 0.004 | FALSE |
| Calcium | 0.001 | FALSE | 0.003 | FALSE |
| Chloride | 0.009 | FALSE | < 0.001 | FALSE |
| Creatinine | < 0.001 | FALSE | < 0.001 | FALSE |
| Hematocrit | 0.002 | FALSE | < 0.001 | FALSE |
| Hemoglobin | 0.008 | FALSE | < 0.001 | FALSE |
| Platelets | 0.008 | FALSE | 0.001 | FALSE |
| Potassium | < 0.001 | FALSE | 0.045 | FALSE |
| Glucose | < 0.001 | FALSE | 0.001 | FALSE |
| Bicarbonate | 0.002 | FALSE | 0.006 | FALSE |
| Sodium | 0.002 | FALSE | < 0.001 | FALSE |
| WBC | 0.005 | FALSE | < 0.001 | FALSE |
| INR | < 0.001 | FALSE | < 0.001 | FALSE |
| PTT | < 0.001 | FALSE | < 0.001 | FALSE |

MIMIC, Medical Information Mart for Intensive Care; eICU-CRD, eICU Collaborative Research Database; BMI, body mass index; GCS, Glasgow Coma Score; APSIII, Acute Physiological Scores II; WBC, white blood cells; BUN, blood urea nitrogen; INR, International Normalized Ratio; PTT, part prothrombin time.
