# Supplementary material for: Influence of age-adjusted shock index trajectories on 30-day mortality for critical patients with septic shock
Source: Front Med (Lausanne). 2025 May 9;12:1534706. doi: 10.3389/fmed.2025.1534706 (PMC12098450; doi:10.3389/fmed.2025.1534706)
Supplement: Supplementary file 1 [file Data_Sheet_1.zip › Supplementary Material/Supplement Table 3.docx]

**Supplement Table 3. Results of collinearity diagnosis.**

| Variables | MIMIC-Ⅳ | eICU-CRD |
| --- | --- | --- |
|  | VIF | VIF |
| Age | 1.500 | 1.075 |
| BMI | 1.186 | 1.136 |
| GCS | 1.318 | 1.393 |
| ASPIII | 3.237 | 1.559 |
| Urine output | 1.513 | 1.007 |
| BUN | 2.343 | 1.492 |
| Calcium | 1.235 | 1.082 |
| Chloride | 1.505 | 1.048 |
| Creatinine | 2.642 | 1.463 |
| Hematocrit | 1.661 | 1.171 |
| Hemoglobin | 1.414 | 1.161 |
| Platelets | 1.408 | 1.093 |
| Potassium | 1.398 | 1.190 |
| Glucose | 1.107 | 1.042 |
| Bicarbonate | 2.372 | 1.114 |
| Sodium | 4.298 | 1.012 |
| WBC | 1.181 | 1.111 |
| INR | 1.348 | 1.352 |
| PPT | 1.283 | 1.299 |

VIF: variance inflation factor; MIMIC, Medical Information Mart for Intensive Care; eICU-CRD, eICU Collaborative Research Database; GCS: Glasgow Coma Score; APSIII: Acute Physiological Scores II; WBC: white blood cells; BUN: blood urea nitrogen; INR: International Normalized Ratio; PTT: part prothrombin time.
