# Supplementary material for: Influence of age-adjusted shock index trajectories on 30-day mortality for critical patients with septic shock
Source: Front Med (Lausanne). 2025 May 9;12:1534706. doi: 10.3389/fmed.2025.1534706 (PMC12098450; doi:10.3389/fmed.2025.1534706)
Supplement: Supplementary file 1 [file Data_Sheet_1.zip › Supplementary Material/Supplement Table 4.docx]

**Supplement Table 4. Baseline characteristics of three classes after IPTW in the derivation cohort.**

| Variables | IPTW | | | | Stabilized IPTW | | | | XGBoost | | | |
| --- | --- | --- | --- | --- | --- | --- | --- | --- | --- | --- | --- | --- |
|  | Class1 | Class2 | Class3 | *P* | Class1 | Class2 | Class3 | *P* | Class1 | Class2 | Class3 | *P* |
| Total (n, %) | 2439.9 (83.5) | 291.4 (10.0) | 189.4 (6.5) |  | 2312.1 (83.5) | 276.1 (10.0) | 179.5 (6.5) |  | 2543.5 (59.0) | 1050.1 (24.4) | 715.8 (16.6) |  |
| Age (years) | 67.9 (15.6) | 73.1 (11.4) | 58.2 (25.2) | < 0.001 | 66.85 (15.6) | 81.5 (9.1) | 84.3 (9.8) | < 0.001 | 67.8 (15.6) | 74.2 (11.9) | 72.3 (16.7) | < 0.001 |
| Gender (n, %) |  |  |  | 0.770 |  |  |  | 0.837 |  |  |  | 0.497 |
| Male | 1330.7 (54.8) | 28.4 (38.9) | 32.5 (73.6) |  | 1283.2 (55.5) | 140.4 (50.9) | 94.6 (52.7) |  | 1396.9 (54.9) | 503.9 (48.0) | 421.4 (58.9) |  |
| Female | 1095.4 (45.2) | 44.6 (61.1) | 11.6 (26.4) |  | 1028.9 (44.5) | 135.7 (49.1) | 84.9 (47.3) |  | 1146.6 (45.1) | 546.2 (52.0) | 294.5 (41.1) |  |
| Ethnicity (n, %) |  |  |  | 0.710 |  |  |  | 0.581 |  |  |  | 0.546 |
| White | 1663.0 (68.5) | 55.8 (76.4) | 23.4 (53.1) |  | 1585.4 (68.6) | 172.4 (62.4) | 116.0 (64.6) |  | 1745.6 (68.6) | 748.6 (71.3) | 411.0 (57.4) |  |
| Black | 338.8 (14.0) | 9.5 (12.9) | 16.3 (37.0) |  | 319.3 (13.8) | 49.8 (18.0) | 14.2 (7.9) |  | 350.4 (13.8) | 164.6 (15.7) | 147.9 (20.7) |  |
| Other | 424.3 (17.5) | 7.8 (10.7) | 4.4 (9.9) |  | 407.5 (17.6) | 53.9 (19.5) | 49.3 (27.5) |  | 447.5 (17.6) | 136.8 (13.0) | 156.9 (21.9) |  |
| BMI (kg/m^2^) | 28.53 (7.42) | 25.26 (4.79) | 28.38 (7.69) | < 0.001 | 28.53 (7.42) | 25.26 (4.79) | 28.38 (7.69) | < 0.001 | 28.44 (7.38) | 26.45 (6.55) | 29.34 (6.78) | 0.133 |
| Unit type (n, %) |  |  |  | 0.648 |  |  |  | 0.682 |  |  |  | 0.856 |
| MICU/SICU | 2079.2 (85.7) | 58.0 (79.4) | 38.8 (88.0) |  | 1986.9 (85.9) | 236.7 (85.7) | 160.6 (89.5) |  | 2182.1 (85.8) | 859.0 (81.8) | 602.0 (84.1) |  |
| CCU | 132.3 (5.5) | 10.8 (14.8) | 2.9 (6.5) |  | 123.6 (5.3) | 8.6 (3.1) | 3.1 (1.7) |  | 138.1 (5.4) | 94.7 (9.0) | 54.7 (7.6) |  |
| Others | 214.5 (8.8) | 4.3 (5.8) | 2.4 (5.5) |  | 201.6 (8.7) | 30.8 (11.1) | 15.8 (8.8) |  | 223.3 (8.8) | 96.4 (9.2) | 59.1 (8.3) |  |
| GCS | 13.8 (1.8) | 13.5 (1.8) | 14.1 (1.5) | 0.028 | 13.8 (1.8) | 13.4 (2.1) | 12.5 (2.2) | 0.033 | 13.8 (1.8) | 13.9 (1.6) | 13.8 (1.7) | 0.868 |
| ASPIII | 64.3 (24.7) | 68.0 (23.9) | 75.64 (23.91) | < 0.001 | 62.4 (23.4) | 95.2 (35.5) | 104.4 (24.9) | < 0.001 | 63.9 (24.3) | 74.4 (29.1) | 75.8 (25.1) | < 0.001 |
| Vasopressor (n, %) |  |  |  | < 0.001 |  |  |  | 0.219 |  |  |  | 0.348 |
| No | 2301.5 (94.9) | 70.0 (95.9) | 34.0 (77.1) |  | 2199.7 (95.1) | 251.3 (91.0) | 174.7 (97.3) |  | 2416.6 (95.0) | 964.2 (91.8) | 639.8 (89.4) |  |
| Yes | 124.6 (5.1) | 3.0 (4.1) | 10.1 (22.9) |  | 112.4 (4.9) | 24.9 (9.0) | 4.8 (2.7) |  | 126.9 (5.0) | 85.9 (8.2) | 76.0 (10.6) |  |
| Ventilation (n, %) |  |  |  | 0.368 |  |  |  | 0.336 |  |  |  | 0.367 |
| No | 273.4 (11.3) | 5.2 (7.2) | 1.6 (3.7) |  | 272.3 (11.8) | 10.4 (3.8) | 29.8 (16.6) |  | 288.2 (11.3) | 87.6 (8.3) | 33.9 (4.7) |  |
| Yes | 2152.7 (88.7) | 67.8 (92.8) | 42.5 (96.3) |  | 2039.8 (88.2) | 265.7 (96.2) | 149.7 (83.4) |  | 2255.3 (88.7) | 962.5 (91.7) | 681.9 (95.3) |  |
| Urine output (ml) | 1483.9 (1143.9) | 1190.28 (912.38) | 1380.87 (900.68) | < 0.001 | 1527.2 (1147.6) | 1029.5 (1075.4) | 543.8 (699.7) | < 0.001 | 1486.6 (1142.1) | 1141.9 (918.7) | 1340.9 (1110.6) | 0.006 |
| BUN (mg/dL) | 35.99 (22.72) | 38.44 (23.32) | 31.16 (20.44) | 0.261 | 35.7 (22.9) | 37.5 (18.0) | 47.2 (20.0) | 0.035 | 36.1 (22.8) | 36.9 (21.8) | 34.8 (19.2) | 0.870 |
| Calcium (mmol/l) | 7.85 (0.75) | 7.91 (0.73) | 8.06 (0.88) | 0.003 | 7.9 (0.7) | 7.3 (0.7) | 7.9 (1.1) | < 0.001 | 7.9 (0.8) | 7.8 (0.8) | 7.8 (0.8) | 0.727 |
| Chloride (mEq/l) | 104.98 (6.80) | 105.86 (5.57) | 105.21 (5.24) | 0.157 | 104.9 (6.8) | 107.1 (5.7) | 109.4 (6.9) | 0.001 | 105.0 (6.8) | 105.9 (5.8) | 105.0 (6.0) | 0.431 |
| Creatinine (g/dl) | 1.83 (1.21) | 1.78 (1.12) | 2.02 (1.20) | 0.135 | 1.8 (1.2) | 1.7 (0.9) | 2.2 (1.0) | 0.151 | 1.84 (1.22) | 1.74 (1.07) | 2.13 (1.21) | 0.332 |
| Glucose (mg/dl) | 144.16 (52.26) | 137.31 (43.81) | 166.87 (57.97) | 0.331 | 143.7 (51.7) | 160.8 (62.4) | 160.5 (65.9) | 0.146 | 144.10 (52.1) | 142.34 (51.74) | 153.19 (50.67) | 0.565 |
| Bicarbonate (mEq/l) | 20.97 (4.56) | 21.08 (3.49) | 20.54 (4.30) | 0.001 | 21.1 (4.5) | 18.2 (3.7) | 17.4 (5.7) | < 0.001 | 20.9 (4.6) | 20.5 (3.9) | 21.1 (5.9) | 0.639 |
| Hematocrit (g/dl) | 31.68 (5.55) | 30.65 (4.99) | 32.41 (4.54) | 0.163 | 31.7 (5.6) | 31.4 (4.9) | 37.8 (7.2) | 0.002 | 31.7 (5.5) | 30.2 (5.5) | 32.2 (5.6) | 0.084 |
| Hemoglobin (g/dl) | 10.30 (1.85) | 9.86 (1.54) | 10.67 (1.59) | 0.526 | 10.3 (1.9) | 10.2 (1.6) | 11.3 (1.9) | 0.066 | 10.3 (1.8) | 9.8 (1.7) | 10.3 (1.8) | 0.081 |
| Platelets (10^9^/l) | 208.37 (118.65) | 234.75 (112.79) | 222.52 (111.66) | 0.263 | 206.9 (118.4) | 237.2 (136.3) | 239.4 (134.5) | 0.202 | 208.2 (118.6) | 222.3 (127.7) | 228.1 (117.6) | 0.404 |
| Potassium (mmol/l) | 4.23 (0.67) | 4.25 (0.58) | 4.28 (0.66) | 0.356 | 4.2 (0.7) | 4.3 (0.6) | 4.5 (0.8) | 0.213 | 4.2 (0.7) | 4.2 (0.6) | 4.2 (0.6) | 0.884 |
| WBC (10^9^/l) | 14.52 (8.00) | 16.93 (7.87) | 15.38 (7.55) | 0.014 | 14.3 (7.9) | 16.3 (10.6) | 17.2 (8.8) | 0.123 | 14.5 (8.0) | 15.8 (8.8) | 16.8 (8.6) | 0.139 |
| Sodium (mmol/l) | 137.84 (5.16) | 139.50 (5.40) | 139.09 (3.50) | 0.332 | 137.8 (5.1) | 136.8 (4.8) | 141.2 (5.0) | 0.005 | 137.8 (5.1) | 138.7 (5.2) | 138.8 (4.1) | 0.223 |
| INR | 1.66 (0.58) | 1.68 (0.49) | 1.91 (0.71) | 0.030 | 1.7 (0.6) | 1.8 (0.6) | 1.9 (0.7) | 0.033 | 1.7 (0.6) | 1.72 (0.52) | 1.7 (0.7) | 0.592 |
| PTT (seconds) | 40.24 (13.12) | 42.61 (13.23) | 41.22 (12.12) | 0.015 | 39.9 (12.9) | 42.8 (10.9) | 52.4 (14.3) | < 0.001 | 40.2 (13.1) | 41.9 (12.8) | 40.8 (13.9) | 0.551 |

XGBoost, Extreme Gradient Boosting; IPTW, inverse probability of treatment weighting; BMI: body mass index; MICU: medical intensive care unit; SICU: surgical intensive care unit; CCU: coronary care unit; GCS, Glasgow Coma Score; APSIII, Acute Physiological Scores II; WBC, white blood cells; BUN, blood urea nitrogen; INR, International Normalized Ratio; PTT, part prothrombin time.
