# Supplementary material for: Influence of age-adjusted shock index trajectories on 30-day mortality for critical patients with septic shock
Source: Front Med (Lausanne). 2025 May 9;12:1534706. doi: 10.3389/fmed.2025.1534706 (PMC12098450; doi:10.3389/fmed.2025.1534706)
Supplement: Supplementary file 1 [file Data_Sheet_1.zip › Supplementary Material/Supplement Table 5.docx]

**Supplement Table 5. Baseline characteristics of three classes after IPTW in the validation cohort.**

| Variables | IPTW | | | | Stabilized IPTW | | | | XGBoost | | | |
| --- | --- | --- | --- | --- | --- | --- | --- | --- | --- | --- | --- | --- |
|  | Class 1 | Class 2 | Class 3 | *P* | Class 1 | Class 2 | Class 3 | *P* | Class 1 | Class 2 | Class 3 | *P* |
| Total (n, %) | 2017.7 (79.2) | 304.9 (12.0) | 223.1 (8.8) |  | 1849.0 (79.2) | 279.4 (12.0) | 204.5 (8.8) |  | 2146.6 (62.4) | 712.7 (20.7) | 579.2 (16.9) |  |
| Age (years) | 63.2 (15.39) | 80.3 (7.58) | 79.0 (7.17) | < 0.001 | 63.2 (15.39) | 80.2 (7.58) | 79.0 (7.17) | < 0.001 | 64.8 (15.36) | 71.2 (11.39) | 68.3 (11.95) | < 0.001 |
| Gender (n, %) |  |  |  | 0.002 |  |  |  | 0.002 |  |  |  | 0.026 |
| Male | 1076.4 (53.3) | 128.9 (42.3) | 60.2 (27.0) |  | 986.4 (53.3) | 118.1 (42.3) | 55.1 (27.0) |  | 1119.8 (52.2) | 404.5 (56.8) | 192.2 (33.2) |  |
| Female | 941.3 (46.7) | 176.0 (57.7) | 163.0 (73.0) |  | 862.6 (46.7) | 161.3 (57.7) | 149.4 (73.0) |  | 1026.8 (47.8) | 308.2 (43.2) | 387.0 (66.8) |  |
| Ethnicity (n, %) |  |  |  | 0.938 |  |  |  | 0.931 |  |  |  | 0.662 |
| White | 1692.5 (83.9) | 261.6 (85.8) | 183.8 (82.4) |  | 1551.0 (83.9) | 239.8 (85.8) | 168.5 (82.4) |  | 1808.2 (84.2) | 633.5 (88.9) | 453.3 (78.3) |  |
| Black | 179.9 (8.9) | 21.2 (6.9) | 25.3 (11.3) |  | 164.8 (8.9) | 19.4 (6.9) | 23.2 (11.3) |  | 187.5 (8.7) | 39.7 (5.6) | 66.4 (11.5) |  |
| Other | 145.3 (7.2) | 22.1 (7.2) | 14.0 (6.3) |  | 133.2 (7.2) | 20.3 (7.2) | 12.9 (6.3) |  | 150.9 (7.0) | 39.5 (5.5) | 59.5 (10.3) |  |
| BMI (kg/m^2^) | 28.8 (8.9) | 28.8 (8.6) | 25.3 (6.4) | 0.005 | 28.8 (9.0) | 28.9 (8.6) | 25.3 (6.3) | 0.005 | 28.8 (8.9) | 29.2 (10.0) | 27.6 (7.4) | 0.620 |
| Unit type (n, %) |  |  |  | 0.204 |  |  |  | 0.204 |  |  |  | 0.700 |
| MICU/SICU | 1683.5 (83.4) | 238.9 (78.4) | 164.4 (73.7) |  | 1542.8 (83.4) | 219.0 (78.4) | 150.7 (73.7) |  | 1789.3 (83.4) | 607.3 (85.2) | 479.0 (82.7) |  |
| CCU | 226.9 (11.2) | 44.9 (14.7) | 49.9 (22.4) |  | 207.9 (11.2) | 41.1 (14.7) | 45.8 (22.4) |  | 243.0 (11.3) | 53.7 (7.5) | 82.0 (14.2) |  |
| Others | 107.3 (5.3) | 21.1 (6.9) | 8.8 (3.9) |  | 98.3 (5.3) | 19.3 (6.9) | 8.1 (3.9) |  | 114.3 (5.3) | 51.7 (7.3) | 18.1 (3.1) |  |
| GCS | 12.4 (3.6) | 10.97 (4.2) | 10.38 (4.7) | 0.002 | 12.3 (3.5) | 10.97 (4.2) | 10.38 (4.7) | 0.001 | 12.3 (3.6) | 11.5 (4.0) | 11.7 (3.9) | 0.460 |
| ASPIII | 64.9 (27.5) | 93.3 (29.1) | 91.66 (30.1) | < 0.001 | 64.9 (27.5) | 93.2 (29.1) | 91.66 (30.0) | < 0.001 | 66.8 (28.2) | 71.90 (26.97) | 72.73 (24.7) | 0.032 |
| Vasopressor (n, %) |  |  |  | < 0.001 |  |  |  | < 0.001 |  |  |  | 0.104 |
| No | 832.8 (41.3) | 51.5 (16.8) | 23.6 (10.8) |  | 763.2 (41.3) | 46.9 (16.8) | 23.1 (11.3) |  | 838.1 (39.0) | 224.5 (31.5) | 130.0 (22.4) |  |
| Yes | 1184.9 (58.7) | 254.3 (83.2) | 195.4 (89.2) |  | 1085.8 (58.7) | 232.5 (83.2) | 181.4 (88.7) |  | 1308.5 (61.0) | 488.2 (68.5) | 449.2 (77.6) |  |
| Ventilation (n, %) |  |  |  | < 0.001 |  |  |  | < 0.001 |  |  |  | 0.331 |
| No | 1315.3 (65.2) | 173.3 (56.7) | 78.0 (35.6) |  | 1205.3 (65.2) | 158.6 (56.8) | 72.0 (35.2) |  | 1370.5 (63.8) | 394.3 (55.3) | 297.0 (51.3) |  |
| Yes | 702.4 (34.8) | 132.5 (43.3) | 141.0 (64.4) |  | 643.7 (34.8) | 120.8 (43.2) | 132.5 (64.8) |  | 776.1 (36.2) | 318.4 (44.7) | 282.1 (48.7) |  |
| Urine output (ml) | 287.1 (351.0) | 307.0 (441.2) | 287.3 (290.3) | 0.940 | 287.1 (350.9) | 307.0 (441.2) | 287.3 (290.2) | 0.940 | 284.7 (348.5) | 298.97 (418.3) | 248.95 (223.5) | 0.348 |
| BUN (mg/dL) | 32.4 (24.3) | 37.09 (16.6) | 32.25 (20.1) | 0.058 | 32.35 (24.3) | 37.09 (16.6) | 32.25 (20.1) | 0.058 | 32.9 (24.3) | 33.31 (17.9) | 31.7 (21.5) | 0.988 |
| Calcium (mmol/l) | 7.7 (0.9) | 7.7 (0.7) | 7.4 (0.9) | 0.145 | 7.6 (0.9) | 7.7 (0.7) | 7.4 (0.9) | 0.145 | 7.7 (0.9) | 7.6 (0.9) | 7.5 (1.1) | 0.720 |
| Chloride (mEq/l) | 53.7 (42.5) | 54.5 (42.2) | 76.6 (46.0) | 0.015 | 53.7 (42.5) | 54.5 (42.2) | 76.6 (46.0) | 0.015 | 54.3 (42.7) | 56.8 (40.5) | 60.2 (45.0) | 0.832 |
| Creatinine (g/dl) | 2.0 (1.6) | 2.0 (1.1) | 2.2 (1.1) | 0.424 | 2.0 (1.6) | 2.0 (1.1) | 2.3 (1.2) | 0.424 | 2.1 (1.6) | 2.1 (1.3) | 1.8 (1.0) | 0.634 |
| Glucose (mg/dl) | 119.5 (67.6) | 126.4 (66.4) | 144.9 (59.5) | 0.014 | 119.4 (67.6) | 126.4 (66.3) | 144.8 (59.4) | 0.014 | 120.9 (68.7) | 120.7 (61.8) | 121.5 (61.2) | 0.996 |
| Bicarbonate (mEq/l) | 19.4 (6.7) | 15.4 (6.7) | 15.5 (6.6) | < 0.001 | 19.3 (6.6) | 15.3 (6.6) | 15.5 (6.6) | < 0.001 | 19.1 (6.7) | 19.6 (6.9) | 19.0 (6.4) | 0.930 |
| Hematocrit (g/dl) | 28.29 (9.3) | 25.59 (10.6) | 34.41 (8.5) | < 0.001 | 28.29 (9.3) | 25.59 (10.6) | 34.41 (8.5) | < 0.001 | 28.3 (9.3) | 28.7 (9.2) | 27.7 (11.1) | 0.793 |
| Hemoglobin (g/dl) | 8.5 (3.6) | 8.6 (4.0) | 10.6 (3.4) | 0.005 | 8.5 (3.6) | 8.5 (4.0) | 10.5 (3.4) | 0.005 | 8.5 (3.6) | 8.6 (3.7) | 8.9 (3.3) | 0.743 |
| Platelets (10^9^/l) | 160.3 (118.8) | 159.91 (128.84) | 158.5 (113.9) | 0.990 | 160.3 (118.8) | 159.9 (128.8) | 158.4 (113.9) | 0.990 | 160.1 (119.2) | 162.1 (125.1) | 164.9 (125.9) | 0.944 |
| Potassium (mmol/l) | 4.1 (0.6) | 4.2 (0.6) | 3.9 (0.6) | 0.160 | 4.0 (0.6) | 4.21 (0.6) | 3.97 (0.6) | 0.160 | 4.1 (0.6) | 4.28 (0.70) | 4.1 (0.6) | 0.337 |
| WBC (10^9^/l) | 14.8 (10.6) | 22.7 (17.9) | 11.5 (11.4) | 0.001 | 14.89 (10.6) | 22.78 (17.9) | 11.5 (11.4) | 0.001 | 15.0 (10.7) | 16.7 (11.2) | 14.5 (10.0) | 0.475 |
| Sodium (mmol/l) | 125.8 (32.4) | 122.4 (38.1) | 128.0 (26.1) | 0.741 | 125.84 (32.4) | 122.4 (38.1) | 128.04 (26.1) | 0.741 | 126.0 (32.1) | 125.2 (28.4) | 122.4 (36.5) | 0.871 |
| INR | 1.8 (1.0) | 2.1 (1.1) | 2.5 (2.0) | 0.012 | 1.81 (1.0) | 2.1 (1.1) | 2.5 (2.0) | 0.012 | 1.8 (1.0) | 2.0 (1.2) | 1.9 (1.4) | 0.455 |
| PTT (seconds) | 39.7 (21.6) | 44.7 (31.6) | 41.0 (18.3) | 0.397 | 39.7 (21.6) | 44.7 (31.6) | 41.0 (18.3) | 0.397 | 40.0 (22.1) | 41.3 (26.3) | 36.9 (20.5) | 0.317 |

XGBoost, Extreme Gradient Boosting; IPTW, inverse probability of treatment weighting; BMI: body mass index; MICU: medical intensive care unit; SICU: surgical intensive care unit; CCU: coronary care unit; GCS, Glasgow Coma Score; APSIII, Acute Physiological Scores II; WBC, white blood cells; BUN, blood urea nitrogen; INR, International Normalized Ratio; PTT, part prothrombin time.
