# Supplementary figures and images for: Influence of age-adjusted shock index trajectories on 30-day mortality for critical patients with septic shock
Source: Front Med (Lausanne). 2025 May 9;12:1534706. doi: 10.3389/fmed.2025.1534706 (PMC12098450; doi:10.3389/fmed.2025.1534706)

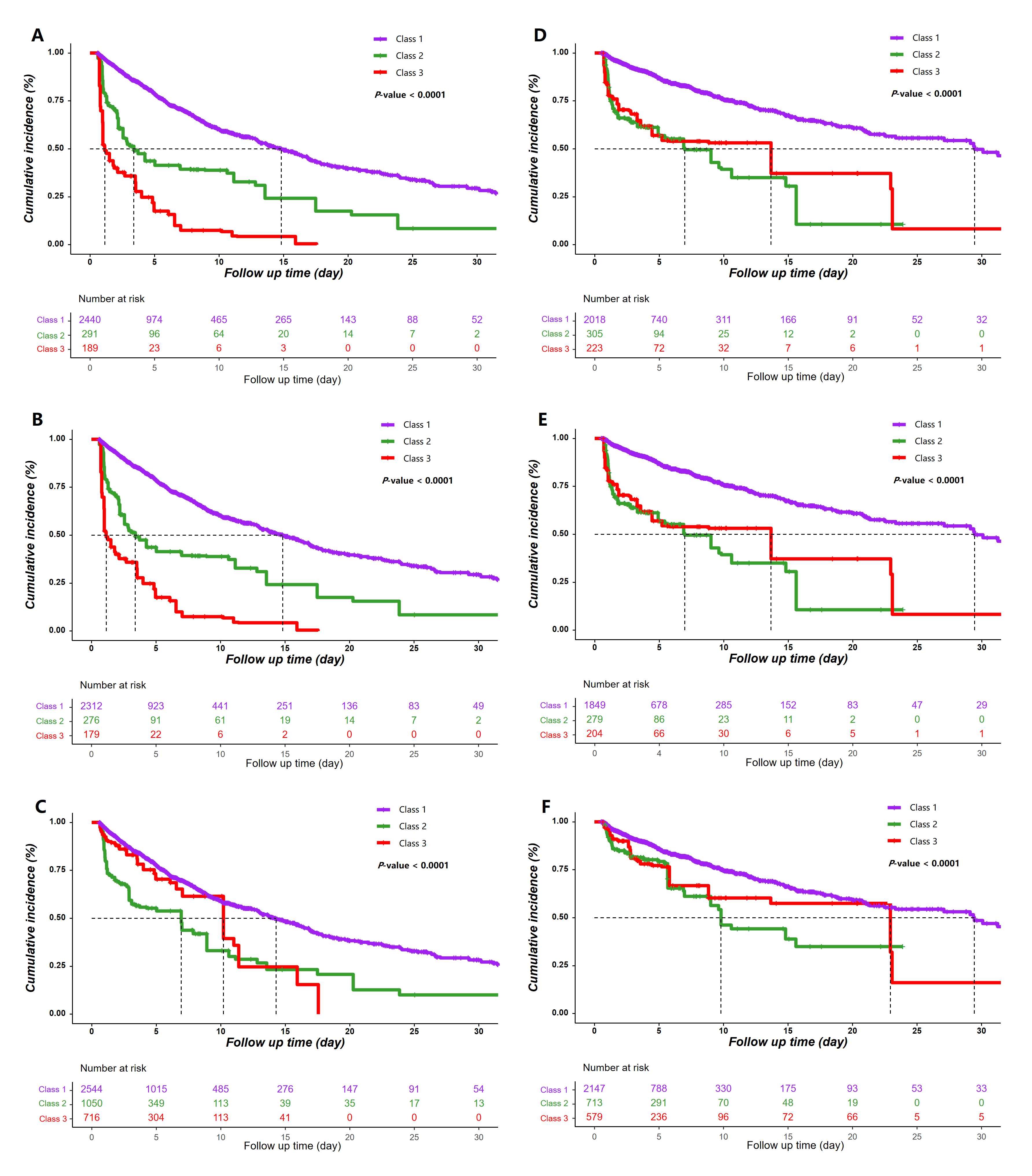

Supplement: Supplementary file 1 [file Data_Sheet_1.zip › Supplementary Material/Supplement Figure 1.tif]
